# Growth of GaN nanowall network on Si (111) substrate by molecular beam epitaxy

**DOI:** 10.1186/1556-276X-7-686

**Published:** 2012-12-27

**Authors:** Aihua Zhong, Kazuhiro Hane

**Affiliations:** 1 Department of Nanomechanics, Tohoku University, Sendai, 980-8579, Japan

**Keywords:** GaN nanowall network, GaN growth on Si substrate, Porous GaN, Hall measurement, N/Ga ratio, TEM

## Abstract

GaN nanowall network was epitaxially grown on Si (111) substrate by molecular beam epitaxy. GaN nanowalls overlap and interlace with one another, together with large numbers of holes, forming a continuous porous GaN nanowall network. The width of the GaN nanowall can be controlled, ranging from 30 to 200 nm by adjusting the N/Ga ratio. Characterization results of a transmission electron microscope and X-ray diffraction show that the GaN nanowall is well oriented along the *C* axis. Strong band edge emission centered at 363 nm is observed in the spectrum of room temperature photoluminescence, indicating that the GaN nanowall network is of high quality. The sheet resistance of the Si-doped GaN nanowall network along the lateral direction was 58 Ω/. The conductive porous nanowall network can be useful for integrated gas sensors due to the large surface area-to-volume ratio and electrical conductivity along the lateral direction by combining with Si micromachining.

## Background

GaN semiconductors exhibit excellent properties in optical devices and high-power/high-frequency electronics, such as light-emitting diodes [1], laser diodes [2], and AlGaN/GaN high-electron mobility transistors [3]. Much attention also has been paid to GaN nanostructures because nanoscale materials, such as nanowires [4], nanotubes [5], and nanorods [6], are dislocation-free and strain-free with a large surface area-to-volume ratio [7,8]. Due to these characteristics, GaN nanostructures exhibit superior performance to conventional planar GaN. An optoelectronic device using GaN nanowires was demonstrated in [9].

Though these GaN nanostructures (nanotube, nanowire, and nanocolumn) are exhibiting promising properties, fabrication of an electronic device based on them is complicated because the separation of nanostructures inhibits electric current from flowing among these nanostructures. In the case of a photo detector based on GaN nanowires, the detector was fabricated on an individual nanowire [10]. Fabrication of an electronic device on an individual nanowire is highly difficult.

Nanowalls are attractive due to their porous surface and material continuity along the lateral direction. Carbon [11,12], ZnO [13,14], and NiO [15] nanowalls have been investigated. Kesaria et al. reported the growth of a GaN nanowall network on a sapphire substrate [16-18]. In these papers, transformation among the GaN nanowall network, GaN nanocolumn, and GaN film is observed by changing the growth condition. On one hand, the width of the GaN nanowall is in nanoscale and, in terms of property, is as good as a separated nanostructure [16]. On the other hand, unlike nanotubes and nanowires, the GaN nanowall network is continuous along the lateral direction. Because of this characteristic, the GaN nanowall network is expected to be fabricated to nanodevices as easily as the GaN film. A gas sensor was fabricated on a ZnO nanowall network using the same technology as film device [19]. Especially, using Si substrate, Si-based micromachining as well as integrated circuit can be applied to an integrated sensor [20].

In this paper, GaN nanowall networks were grown on Si (111) substrate by molecular beam epitaxy (MBE). Growth of GaN on silicon makes it compatible with the most mature silicon-based semiconductor technology. Characterization of the GaN nanowall was carried out. Adjustment of the nanowall width ranging from 30 to 200 nm is achieved by adjusting the N/Ga ratio. Hall mobility and carrier concentration of the Si-doped GaN nanowall network were measured using Hall measurement system.

## Methods

The GaN nanowall network was deposited on Si (111) substrate using a Riber 32 MBE system equipped with a N_2_ RF plasma source (RFS-N/TH, Veeco Instruments Inc., Plainview, NY, USA). The base pressure of the growth chamber is 3.0 × 10^−10^ Torr. The purity of N_2_, Ga, and Al is 99.9999%. A 380-μm-thick Si (111) substrate with a resistivity larger than 5,000 Ω·cm was cleaned in alcohol, followed by standard RCA process. Then, it was dipped in HF:H_2_O = 1:50 for a few seconds to remove the silicon oxide layer on the surface of the Si substrate as well as to form a hydrogen-terminated surface. After cleaning of the substrate, it was immediately blown dry by N_2_ and transferred to a loading chamber for pre-heating at a pressure of 10^−7^ Torr to remove residual hydrocarbons [21].

In our previous work [22], we intentionally nitrided the Si substrate before the growth of GaN, and we observed GaN nanocolumns on this nitrided Si substrate. For the samples shown in this paper, we pre-deposited several monolayers of Al before igniting the N_2_ plasma source to avoid nitridation of the substrate, followed by growth of an about 40-nm-thick AlN buffer layer. Then, ten pairs of AlN (5 nm)/GaN (15 nm) multilayer were grown on the AlN buffer layer. Finally, six GaN samples were grown on the multilayer with various N/Ga ratios from 980 to 180 at 700°C. Adjusting of N/Ga ratio was achieved by changing the temperature of the Ga cell while N_2_ flow was kept constant. The N/Ga ratio is determined by the N flux/Ga flux. For convenience, Ga and N fluxes are given in terms of corresponding beam equivalent pressures measured by a Bayard-Alpert gauge. A Si-doped GaN nanowall network was also grown with a N/Ga ratio of 400 under the same growth procedure. Solid Si effusion cell heated at 1,200°C was used for Si doping.

Field emission scanning electron microscopy (FESEM; S-4500, Hitachi Ltd., Tokyo, Japan), transmission electron microscope (TEM; Hitachi HF 2000, Hitachi Ltd.) and X-ray diffraction (XRD; PW3040/60 X'pert PRO, PANalytical B.V., Almelo, The Netherlands) were used for characterization. A photoluminescence (PL) spectrum analyzer with He-Cd laser (325 nm, 200 mW) as excitation source was also used to investigate the optical property of the GaN nanowall network. Hall parameters of the Si-doped GaN nanowall network were carried out using the Hall measurement system.

## Results and discussion

**Figure 1:**
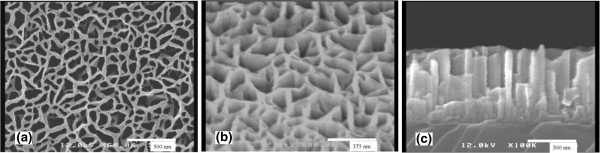
**FESEM images of GaN nanowall network grown with N/Ga ratio 800.** (**a**) Top view, (**b**) 45° tilt, and (**c**) cross section.

From different angles, Figure 1 shows FESEM images of the GaN nanonetwork with a thickness of 500 nm grown on Si (111) substrate with a N/Ga ratio of 800. Though the quality of the image is not very high, it is clear enough to observe the structure. Figure 1a shows the top-view image of the GaN nanonetwork. From Figure 1a, it is observed that GaN nanonetwork is composed of the GaN network line with a width of about 50 nm and large numbers of holes ranging from 50 to 100 nm. These GaN network lines overlap and interlace with one another, together with the large numbers of uniform holes, forming a continuous GaN nanonetwork. Combining the 45° tilt and cross-sectional images shown in Figure 1b,c, it is reasonable to make a conclusion that the network line in Figure 1a corresponds to the GaN nanowall, while the holes correspond to the area where the GaN film was grown. The width of the GaN nanowall is nearly uniform with a value of about 50 nm.

**Figure 2:**
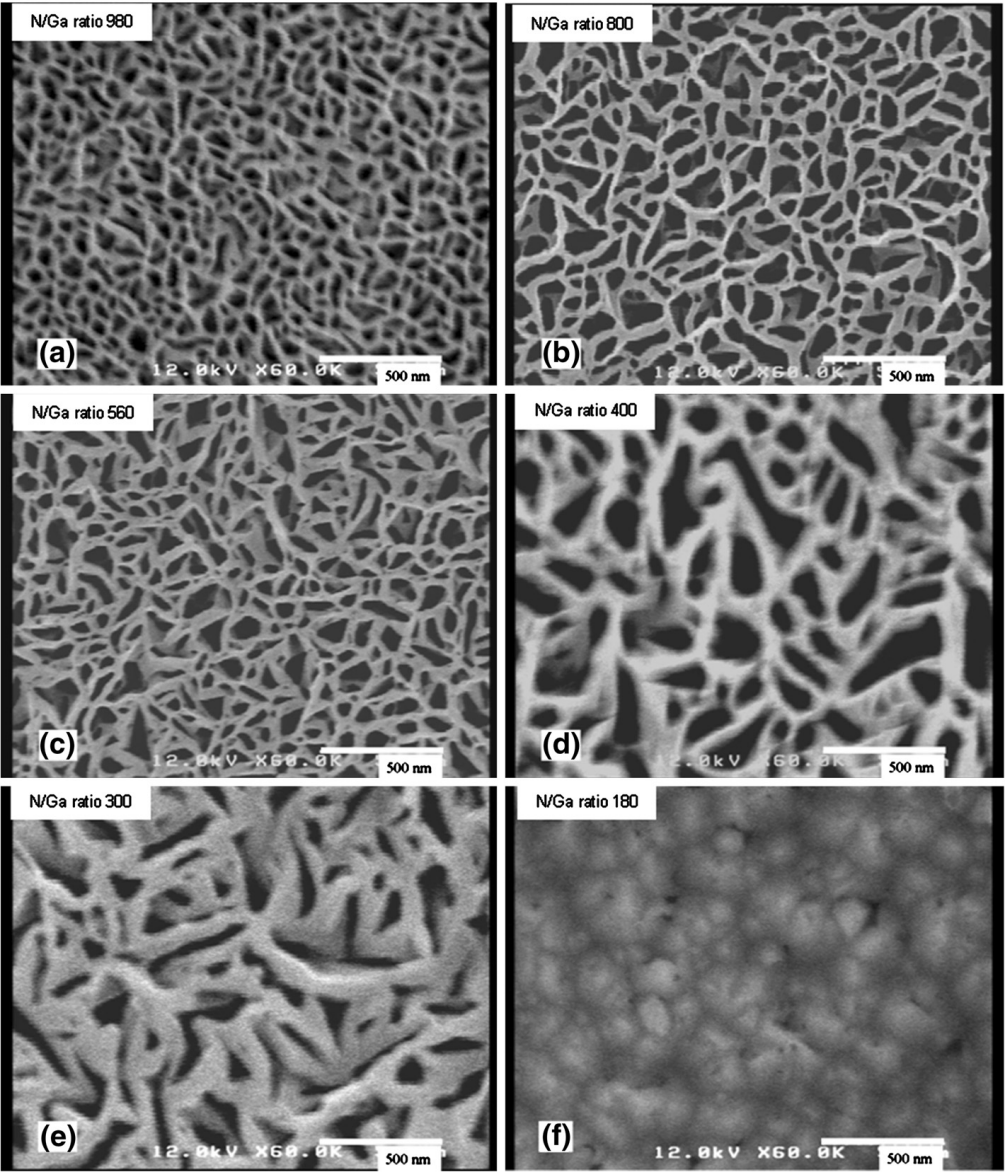
**Top-view FESEM images of GaN grown with different N/Ga ratios.** (**a**) 980, (**b**) 800, (**c**) 560, (**d**) 400, (**e**) 300, and (**f**) 180.

Figure 2 shows the top-view FESEM images of GaN grown with different N/Ga ratios ranging from 980 to 180. For the samples grown with N/Ga ratios larger than 300, they exhibit a nanonetwork structure, and the widths of the GaN nanowalls increase from 30 to 200 nm as the N/Ga ratio decreases. In Figure 2a, the width of the GaN nanowalls is about 30 nm, and the diameter of the holes ranges from 30 to 60 nm. When the N/Ga ratio is decreased to 800 as shown in Figure 2b, the width of the nanowall increases to about 50 nm, and the diameter of the holes also obviously increases to about 100 nm. Further decreasing the N/Ga ratio to 400, the width of the nanowall is increased to about 90 nm as shown in Figure 2d. It is worth noting that when the N/Ga ratio is decreased to 300, most of the surface of the network in Figure 2e is covered by nanowalls with a width of about 200 nm. This kind of nanowall network structure has a large surface area-to-volume ratio, and GaN is continuous in the whole sample in the form of a nanowall. When the N/Ga ratio is 180, however, the network structure disappears and the GaN film is obtained as shown in Figure 2f. No Ga droplet is observed on the whole surface of the sample, together with the appearance of pits, indicating that the GaN film was grown under a nitrogen-rich condition [23].

Therefore, as indicated by Figure 2a,b,c,d,e, the width of the nanowall can be controlled from 30 to 200 nm by adjusting the N/Ga ratio. In a highly nitrogen-rich condition, the Ga adatoms diffuse over a short distance before getting nitrided, promoting three-dimensional nucleation to form the hexagonal GaN nanowall network [16]. With the decrease of the N/Ga ratio, the Ga diffusion distance increases, leading to the change of the nanowall width as shown in Figure 2a,b,c,d,e. When the N/Ga ratio is further decreased to below 180, the nitrogen sticking probability is reduced. Thus, the Ga diffusion distance is increased, forming the GaN film.

**Figure 3:**
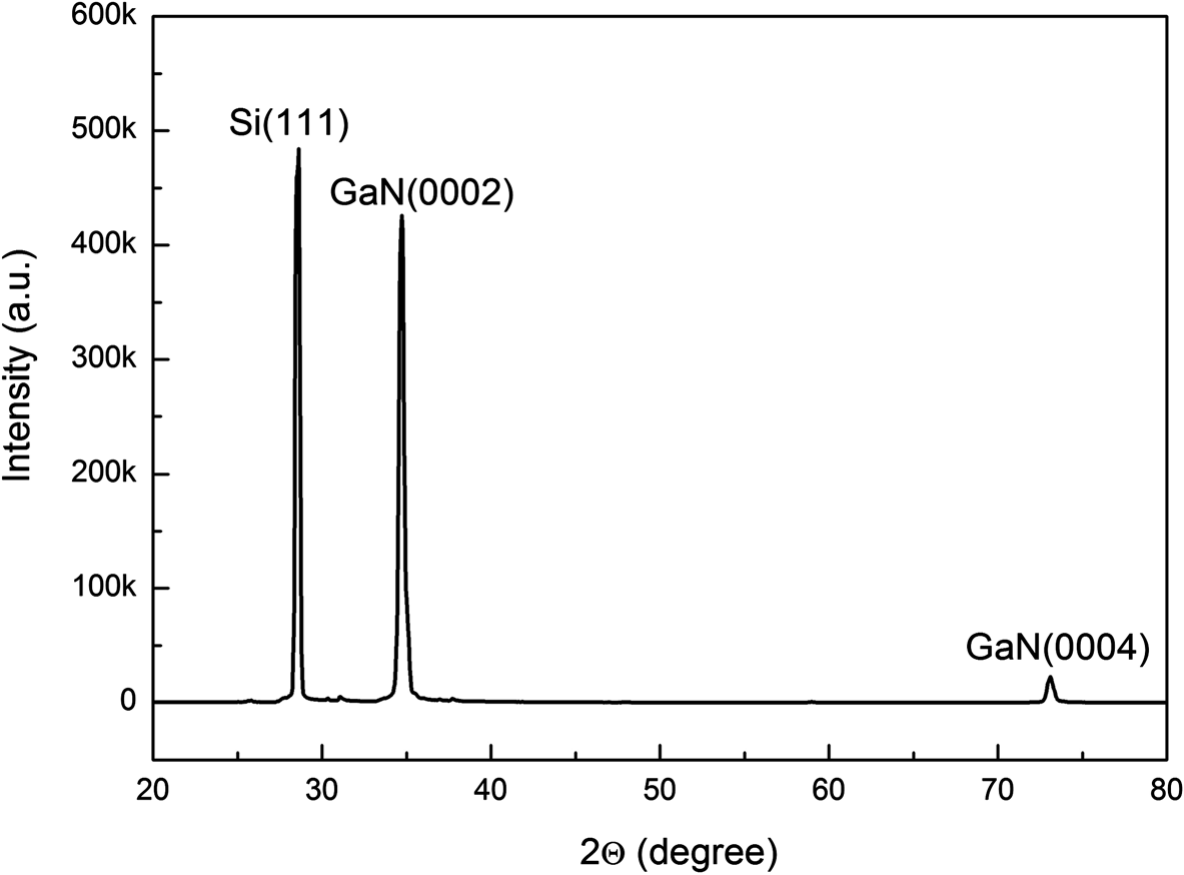
XRD pattern of GaN nanowall network grown with a N/Ga ratio of 560.

**Figure 4:**
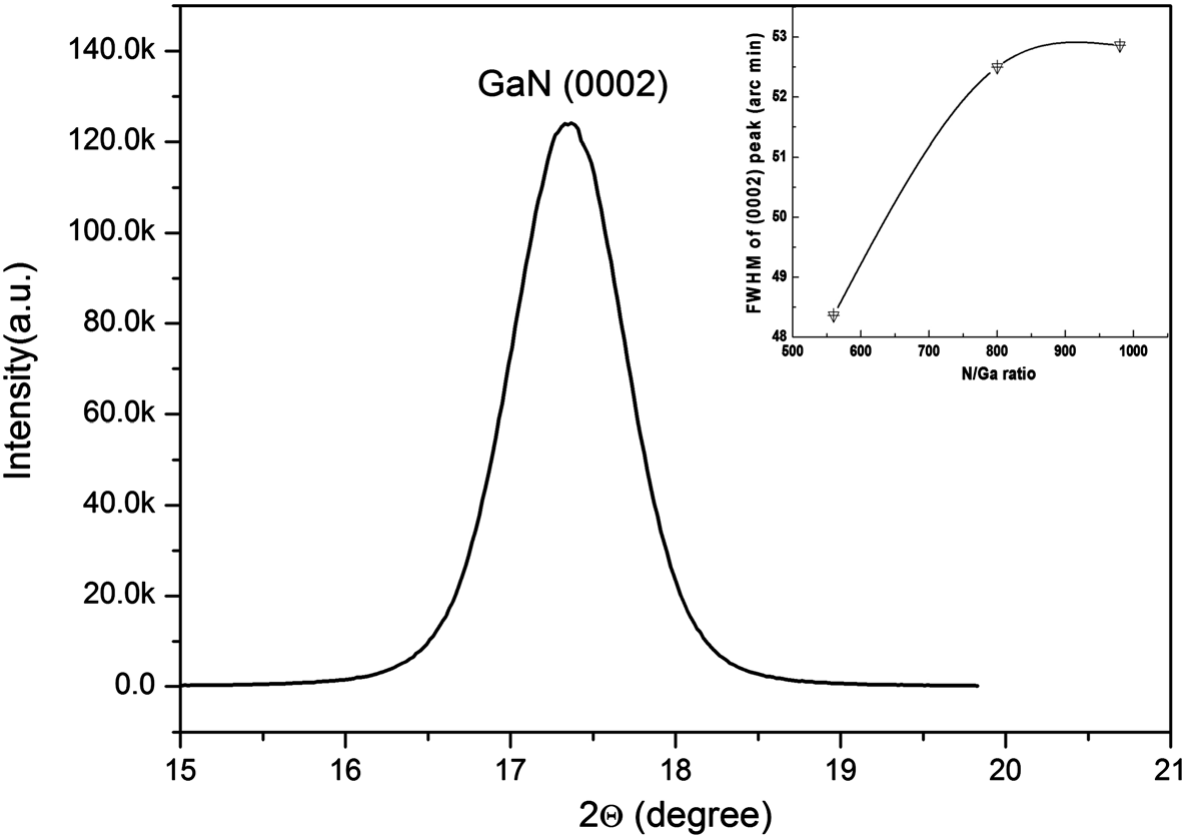
**ω-scan rocking curve of GaN nanowall network grown with a N/Ga ratio of 560.** The inset shows dependence of the FWHM of the GaN (002) peak on N/Ga ratio.

The XRD pattern of GaN grown with a N/Ga ratio of 560 was measured as shown in Figure 3. Only GaN (0002) and GaN (0004) peaks are observed in the XRD pattern. The GaN nanowall network is hexagonal GaN. In addition to the XRD pattern, ω-scan rocking curves of GaN grown with various N/Ga ratios were also measured. Figure 4 shows the ω-scan rocking curve of GaN grown with a N/Ga ratio of 560. The inset exhibits dependence of the full width at half maximum (FWHM) of the GaN (0002) diffraction peak on N/Ga ratios. With the decrease of the N/Ga ratio from 980 to 560, the FWHM decreases from 52.86 to 48.36 arc min. According to Kesaria et al*.*[17], the FWHM of the GaN (0002) diffraction peak grown on sapphire substrate by MBE is observed to decrease from 70 arc min grown at 480°C to 20 arc min grown at 830°C.

**Figure 5:**
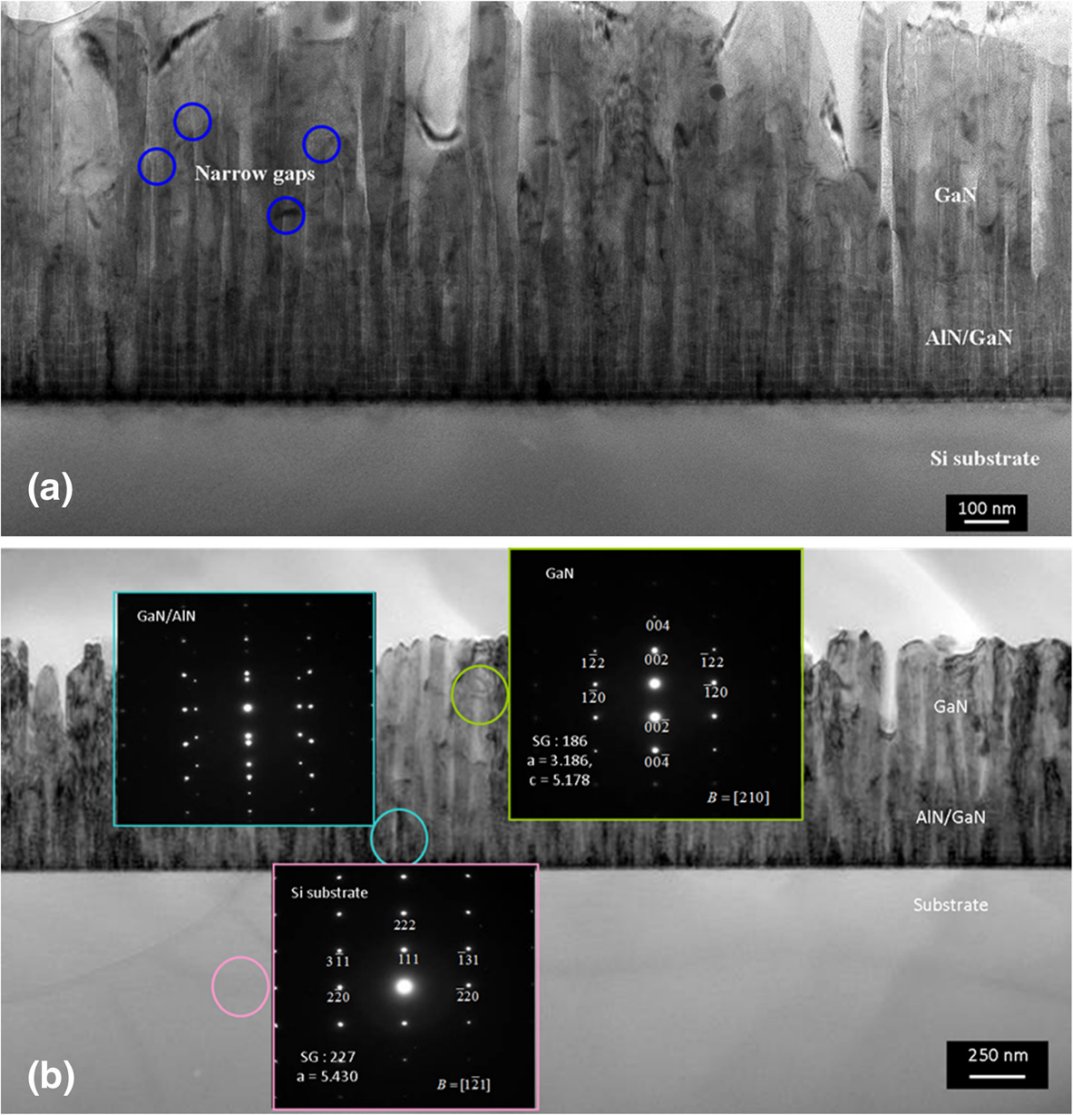
**GaN nanowall network grown with a N/Ga ratio of 400.** (**a**) TEM image and (**b**) electron diffraction patterns.

In order to obtain more details of the GaN nanowall network, TEM was used to characterize the nanowall of GaN grown with a N/Ga ratio of 400. There are some narrow gaps in the GaN nanowall especially at the bottom part, as shown in Figure 5a. As growth continues, these gaps tend to disappear as indicated by blue circles. It seems that the GaN nanowall evolves from the coalescence of nanocolumns. Coalescence of closely spaced GaN nanowires has been reported [24,25]. In addition, the evolution of ZnO nanowires to nanowall was directly observed on an Au-coated sapphire substrate as growth continues [26]. Electron diffraction patterns taken from the Si substrate, AlN/GaN multilayer, and GaN are presented in Figure 5b. The electron diffraction pattern of GaN was measured with an incident beam direction of [1–100]. From these results, it is indicated that the GaN nanowall grows along the *C* axis, vertically aligning with the GaN [0001]//Si [111] direction.

**Figure 6:**
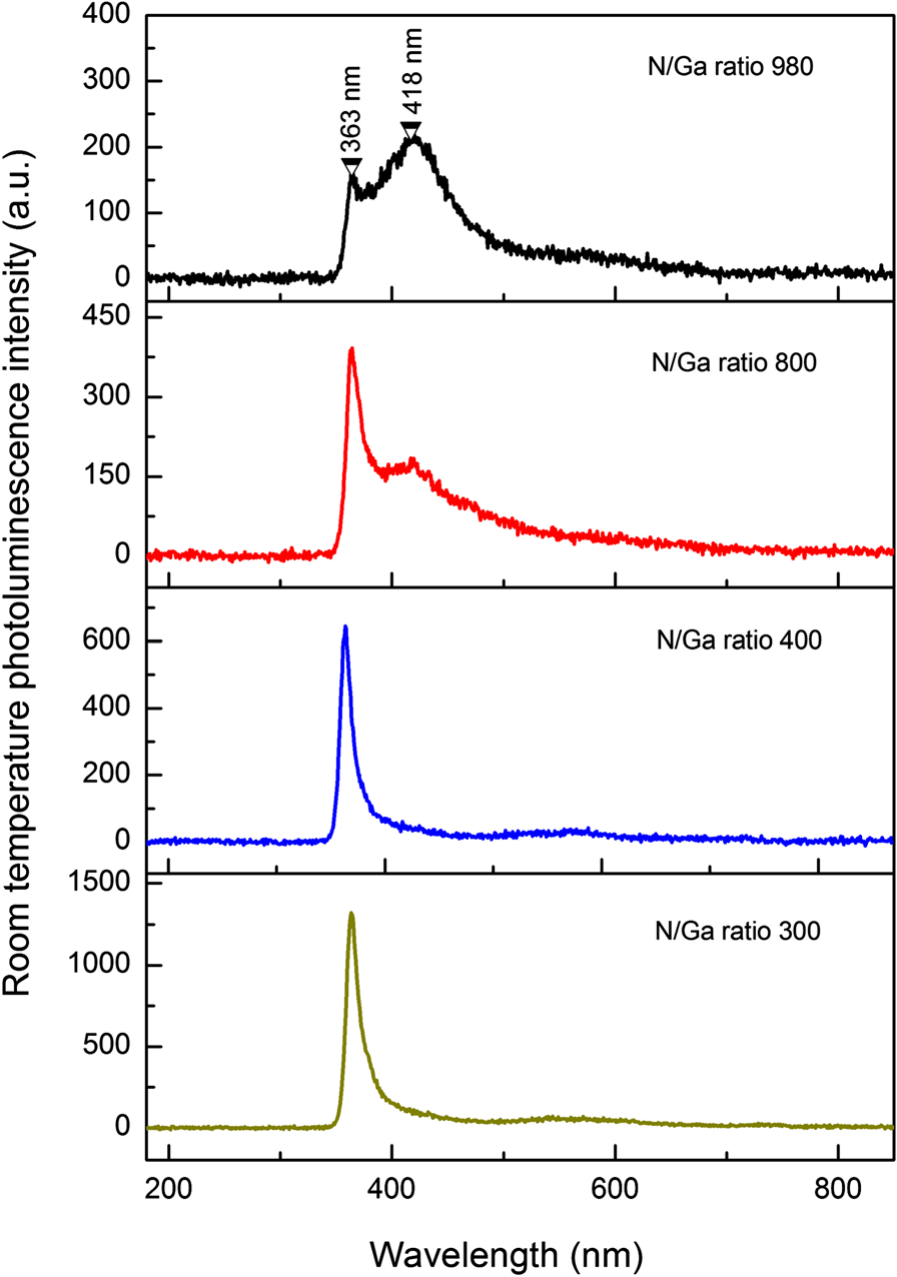
Photoluminescence spectra of GaN nanowall networks grown with different N/Ga ratios.

Room temperature photoluminescence spectra of the GaN network grown with various N/Ga ratios were measured to investigate the influence of the N/Ga ratio on the optical quality of the GaN network, as shown in Figure 6. For the sample grown with a N/Ga ratio of 980, there is a dominant emission peak centered at 418 nm (2.97 eV) together with a weak peak at 363 nm. According to literature [27], 2-/3-, -/2-, and 0/- transition levels of gallium vacancy (*V*_Ga_) are 1.5, 1.0, and 0.5 eV above valence band, respectively. The energy difference of 2.97 eV between the conduction band and 0/- transition level agrees well with the emission peak centered at 418 nm. Therefore, considering that the GaN nanonetwork was grown in a nitrogen-rich condition and that the *V*_Ga_ defect favors to form in this growth condition, the emission peak at 418 nm is attributed to *V*_Ga_.

With the decrease of the N/Ga ratio, the intensity of the emission peak centered at 363 nm increases fast and becomes dominant for the samples grown with N/Ga ratios smaller than 800. Meanwhile, the violet emission at 418 nm decreases gradually with the N/Ga ratio and disappears for the samples grown with N/Ga ratios less than 400. Only the band edge emission at 363 nm with a FWHM of about 12.8 nm is observed in the spectra corresponding to N/Ga ratios of 400 and 300, indicating that GaN networks grown under these conditions are of high quality.

**Figure 7:**
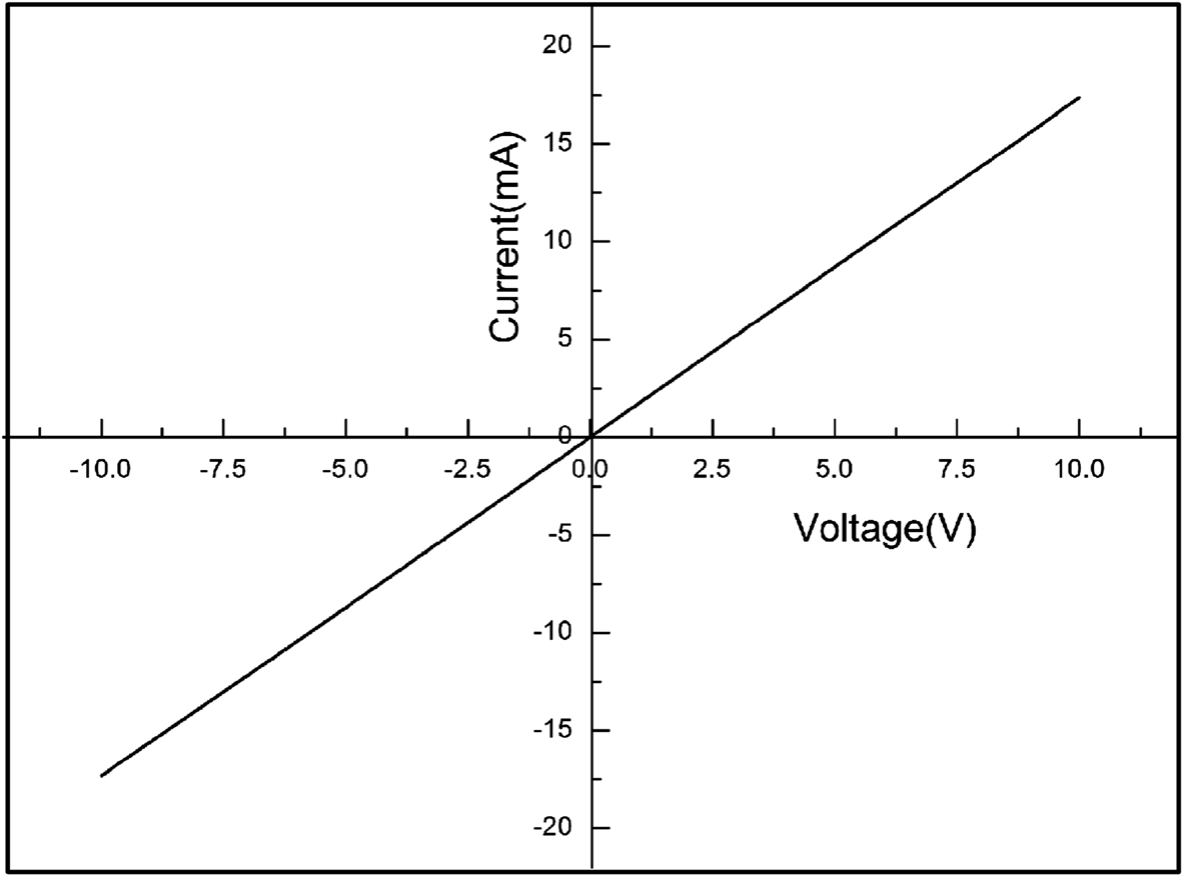
Current–voltage curve of Si-doped GaN nanowall network grown with a N/Ga ratio of 400.

Four ohmic contact Ti (20 nm)/Al (100 nm) electrodes were deposited by electron beam evaporation in the four corners of the 8 × 8 mm Si-doped GaN nanowall network sample grown with a N/Ga ratio of 400 to investigate its electronic properties. The thickness of the Si-doped GaN is 300 nm. The current–voltage curve was measured as shown in Figure 7. The curve indicates that the GaN nanowall network is continuous in the lateral direction for the current in the whole sample, making it as easy as a conventional material to be fabricated to various electronic nanodevices. Hall parameters were measured at 300 K. Electron concentration is 4.6 × 10^19^ cm^−3^ with a sheet resistance of 58 Ω/. Electron mobility at 300 K is 69.7 cm^2^/VS.

Therefore, this nanowall network structure is promising in fields where a large surface/volume ratio is needed, for instance, gas sensors based on surface change after exposing to a particular gas. Compared with separated nanostructures, such as nanowires and nanoparticles, its continuous characteristic along the lateral direction makes it much easier to fabricate to various electronic devices. Moreover, Si substrate is helpful for integrated sensors through the combination with silicon micromachining as well as conventional Si electronics.

## Conclusions

Continuous GaN nanowall network was grown on Si (111) substrate by MBE under N_2_-rich condition. GaN nanowalls overlap and interlace with one another, together with large numbers of holes, forming a continuous GaN nanonetwork. XRD and PL results show that the GaN nanowall network is of high quality. By adjusting the N/Ga ratio, the nanowall width can be varied from 30 to 200 nm. This kind of nanostructure can be fabricated to electronic nanodevices as easily as GaN film. In addition, growth of GaN on silicon makes it compatible with the most mature silicon-based semiconductor technology.

## Competing interests

The authors declare that they have no competing interest.

## Authors’ contributions

AZ carried out the MBE growth and characterization of GaN and drafted the manuscript. KH conceived the study and revised the manuscript. Both authors read and approved the final manuscript.
